# Temporal, but not Directional, Prior Knowledge Shortens Muscle Reflex Latency in Response to Sudden Transition of Support Surface During Walking

**DOI:** 10.3389/fnhum.2016.00029

**Published:** 2016-02-08

**Authors:** Masahiro Shinya, Noritaka Kawashima, Kimitaka Nakazawa

**Affiliations:** ^1^Sports Science Laboratory, Department of Life Sciences, University of TokyoTokyo, Japan; ^2^Department of Rehabilitation for the Movement Functions, Research Institute of National Rehabilitation Center for Persons with Disabilities (NRCD)Tokorozawa, Japan

**Keywords:** anticipation, long-latency reflex, reflex modulation, split-belt treadmill, perturbed walking

## Abstract

The central nervous system takes advantage of prior knowledge about potential upcoming perturbations for modulating postural reflexes. There are two distinct aspects of prior knowledge: spatial and temporal. This study investigated how each of spatial and temporal prior knowledge contributes to the shortening of muscle response latency. Eleven participants walked on a split-belt treadmill and perturbed by sudden acceleration or deceleration of the right belt at right foot contact. Spatial prior knowledge was given by instruction of possible direction (e.g., only acceleration) of upcoming perturbation at the beginning of an experimental session. Temporal prior knowledge was given to subjects by warning tones at foot contact during three consecutive strides before the perturbation. In response to acceleration perturbation, reflexive muscle activity was observed in soleus (SOL) and gastrocnemius (GAS) muscles. Onset latency of the GAS response was shorter (72 ms vs. 58 ms) when subjects knew the timing of the upcoming perturbation, whereas the latency was independent of directional prior knowledge. SOL onset latency (44 ms) was not influenced by directional nor temporal prior knowledge. Although spinal neural circuit that mediates short-latency reflex was not influenced by the prior knowledge, excitability in supra-spinal neural circuit that mediates medium- and long-latency reflex might be enhanced by knowing the timing of the upcoming perturbation.

## Introduction

Stumbling during walking is a common cause of fall-related injuries and thus balance recovery from the perturbation is of critical importance for humans. As the first line of defense, reflexive muscle responses to stumbling perturbation during human walking have been widely studied (Dietz et al., 1984, 1987; Schillings et al., 1996, 1999; Vilensky et al., 1999). Whereas short-latency reflex is hardwired and not flexible, medium and long-latency reflex (latency: ~60 ms), also known as automatic postural responses, have features in between short-latency reflex and voluntary muscle activities: it is automatically triggered by perturbation-related afferent and well-coordinated inter-segmental muscle activation patterns are observed (Dietz et al., 1987; Horak et al., 1997). This task-relevant postural reflex is thought advantageous to maintain balance against a large variety of perturbations that could occur in the real world.

One of the important challenges to the central nervous system is to cope with uncertainty and unpredictability in real-world perturbations. Although it is costly to control the participants’ certainty to upcoming perturbation in laboratory experiments, there have been researches which investigated the effect of prior knowledge on the reflex modulation in response to perturbations during walking (Dietz et al., 1987; Heiden et al., 2006; Siegmund et al., 2006; Shinya and Oda, 2010). For example, the amplitude of tibialis anterior (TA) muscle activity in response to a sudden acceleration of the treadmill belt was reduced if subjects were aware of the potential perturbation (Dietz et al., 1987). For slip perturbations, experience and awareness of the perturbation separately contribute to taking an appropriate landing strategy and reducing startling and co-contractive electromyogram (EMG) responses (Heiden et al., 2006; Siegmund et al., 2006). Not only reflex amplitude, but reflex latency can be shortened by knowing that they might be perturbed by an unexpected hole in a walkway (Shinya and Oda, 2010). These studies clearly suggest the central nervous system takes advantage of prior knowledge about potential upcoming perturbations and modulate walking strategies and reflex patterns.

By having prior knowledge, one can concentrate his/her neuronal resources for dealing with the limited possible perturbation. Therefore, it may matter to what extent of uncertainty one has for generating effective postural responses: when he may be perturbed or by what sort of forces she may be perturbed. However, in these previous experiments, the temporal (timing of the perturbation) and spatial (direction or location of the perturbation) aspects of prior knowledge were not separately controlled, and thus, the effect of each aspect of prior knowledge on the reflexive muscle responses still remain unknown. In this study, we questioned how prior spatial and temporal knowledge might contribute to modulation of reflex in response to perturbations during walking. Specifically, we used a split-belt treadmill where subjects were perturbed with sudden acceleration or deceleration of the right belt at foot contact which is similar to what was used in previous researches (Dietz et al., 1984, 1987). We then manipulated temporal and spatial prior knowledge independently. Reflexive muscle responses to the perturbations were compared between four prior knowledge conditions (the combination of with and without temporal and spatial prior knowledge).

## Experimental Procedures

### Participants

Eleven healthy young males without musculoskeletal or neurological disorders participated in this study. Their mean (± SD) age, height, and weight were 28 ± 5 years, 1.75 ± 0.04 m, and 73 ± 12 kg, respectively. This experiment was not a clinical trial. Informed consent was provided by subjects prior to their participation. The study was conducted in accordance with the Declaration of Helsinki and approved by the NRCD ethics committee.

### Experimental Setup

**Figure 1:**
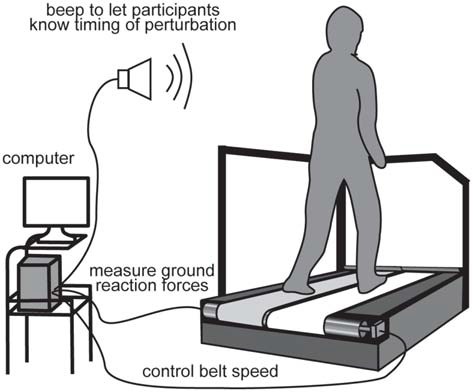
**Experimental setup.** For perturbations, the right belt was suddenly accelerated or decelerated after the right foot touched the belt.

Participants walked and were perturbed on a split-belt treadmill (Figure 1; Bertec, Columbus, OH, USA). The treadmill speed was set to 1.2 m/s throughout the experiment, except during perturbations. We tested acceleration and deceleration perturbations that were induced by accelerating or decelerating the right belt by 0.5 m/s for 400 ms, which occurred immediately after right foot contact. The rate of change in belt speed was set to 20 m/s^2^ for both acceleration and deceleration perturbations. The resultant time interval between right foot contact and the perturbation was 73 ± 3 ms for the acceleration perturbation and 65 ± 5 ms for the deceleration perturbation. The difference in perturbation timing might be due to the mechanical property of the apparatus. The magnitude, timing, and duration of the perturbation were determined through preliminary tests that were large enough to evoke reflexive muscle responses, but small enough not to induce any dangerous injuries and falls. The timing of the perturbations was controlled online using custom-written software in LabVIEW 2010 (National instruments Inc., Austin, TX, USA). The perturbation was induced if the ground reaction force had exceeded 50 N for 10 ms.

### Prior Knowledge Conditions

**Figure 2:**
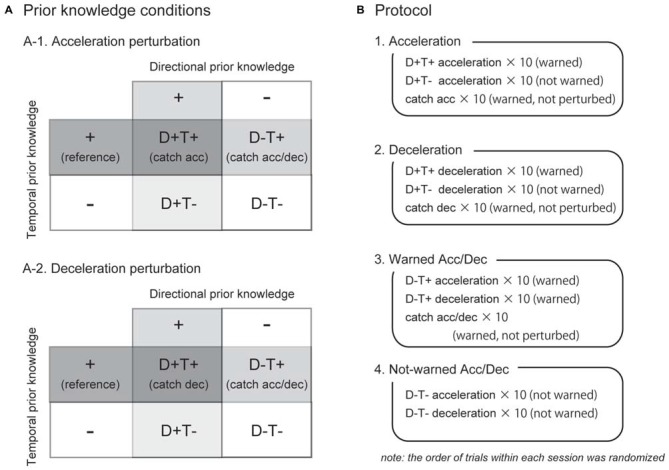
**Experimental conditions and protocol.** As a combination of directional and temporal prior knowledge conditions, four conditions were tested for acceleration and deceleration perturbations **(A)**. For EMG analyses on trials in which the participants knew the timing of an upcoming perturbation (T+), catch trials in which they had been warned but not perturbed were used as reference (written in parenthesis). Each directional prior knowledge condition was tested in separate experimental sessions **(B)**. Before the sessions start, the participants were instructed which perturbation might occur (e.g., the right belt might be accelerated in the session 1). The order of acceleration and deceleration sessions (1 and 2) was counterbalanced among subjects.

The spatial and temporal elements of a participant’s prior knowledge for an upcoming perturbation were separately manipulated. Since we particularly controlled the direction of the perturbation (acceleration or deceleration) as spatial information for the perturbation, henceforth, we used the term “directional prior knowledge” in this study. We tested four prior knowledge conditions as a combination of with or without directional and temporal prior knowledge, abbreviated as D+T+, D+T−, D−T+, and D−T− (Figure 2A). In the D+T− condition, for example, participants knew the direction of the perturbation but did not know the timing of the perturbation. Temporal prior knowledge was given by auditory cues at right foot contact during the three preceding strides before perturbations. The timing of the auditory cue was controlled by the LabVIEW software in the same way as the perturbations. Directional prior knowledge was provided to participants by instructing the direction of the belt perturbation before each experimental session started (e.g., right belt might be accelerated in this session). Note that in directional prior knowledge condition was consistent through one session. Trials with different temporal prior knowledge conditions might be randomly conducted in one session. Each participant performed all the four prior knowledge conditions.

### Protocol

There were four experimental sessions in this study (Figure 2B). The first two sessions were acceleration and deceleration sessions. In these sessions, participants were instructed the direction of the perturbation, thus, trials in these sessions were all D+ conditions. In the acceleration session, participants were instructed that the right belt might accelerate just after right foot contact, with or without warning tones. They also were instructed that there would be catch trials in which they were warned but not perturbed. The session began with 15 s of normal walking followed by 30 test trials. Ten consecutive strides were recorded as a reference of normal walking (normal). The test trials included 10 warned acceleration perturbation trials (D+T+Acc), 10 not-warned acceleration perturbation trials (D+T− Acc), and 10 catch trials (Catch Acc). Similarly, in the deceleration session, 10 warned (D+T+Dec) and 10 not-warned (D+T− Dec) deceleration perturbation trials, in addition to 10 catch trials (Catch Dec), were collected when the participants had prior knowledge that the right belt might be decelerated. The order of acceleration and deceleration sessions was counterbalanced between participants. In this study, since we focused on the effect of prior knowledge on reflexes, practice sessions were performed before the acceleration and deceleration experimental sessions in order to allow participants take a consistent strategy throughout the experimental sessions. In the practice sessions, participants underwent 20 acceleration or deceleration perturbations that were always preceded by warning of auditory cues.

The following two sessions were warned and not-warned Acc/Dec sessions where participants were instructed that the right belt might be accelerated or decelerated so that they did not have directional prior knowledge for upcoming perturbations (i.e., D− conditions). In the warned Acc/Dec session, they were also instructed that all perturbations would be preceded by warning tones, and catch trials would be included. Ten acceleration (D−T+Acc), 10 deceleration (D−T+Dec) perturbation trials, and 10 catch trials (Catch Acc/Dec) were recorded in the warned Acc/Dec session. The last session was a not-warned Acc/Dec session in which the participants had neither of directional and temporal prior knowledge. In this session, 10 not-warned acceleration (D−T− Acc) and 10 deceleration (D−T− Dec) trials were recorded. Note that there was no catch trial in the not-warned Acc/Dec session. Because some participants showed irregular responses in the first 1–3 trials, we used only the last five perturbations for qualitative analyses for each prior knowledge condition. Since no trends were observed in the last five trials, we consider the data reported in this article represents stable strategies for each prior knowledge condition.

### Rationale for Reference Signals

Many researches confirmed that the participants would take proactive strategies for upcoming perturbations if they knew the timing of the perturbations (T+ conditions in this study; Heiden et al., 2006; Siegmund et al., 2006; van der Linden et al., 2007; Nieuwenhuijzen and Duysens, 2007; Shinya and Oda, 2010). In such situations, observed muscle activities after perturbations may include both of reflexive muscle activities and feedforwardly generated muscle activities. For distinguishing perturbation-induced reflexive components from the observed muscle responses, we need to subtract reference muscle activities of the trials where the subjects anticipated the timing of the perturbation but were not actually perturbed: the catch trials (Shinya and Oda, 2010; van der Linden et al., 2007). For D+T+Acc conditions, reference signal was obtained in Catch Acc trials (Figure 2A). Similarly Catch Dec trials were regarded as reference for D+T+Dec condition. For D−T+Acc and D−T+Dec trials, Catch Acc/Dec trials were the reference signals. Note that because of the catch trials, our T+ conditions were conditions where the participants anticipated the perturbations with 50% of probability.

Theoretically, reference muscle activities for the T− perturbations could be different from the normal walking because the participants know that they would be perturbed at some point of the session. Although we observed no significant differences in EMG and ground reaction force signals recorded between the T− conditions and normal walking, we used muscle activities observed in the prior step to the perturbations as references for the T− conditions.

These reference signals were used for the following analyses. First, any differences between catch trials and normal walking were regarded as proactive strategies in the T+ conditions. Second, by subtracting reference signals, we could extract reflexive EMG activities from observed muscle activities in the perturbation trials. Lastly, EMG activity level measured in the reference signals was an index of background alphamotoneuronal excitability level in the corresponding prior knowledge conditions.

### Data Recording

Vertical and horizontal ground reaction forces were recorded separately for the right and left foot. To record muscle reflexes in response to the perturbation, surface EMG was recorded from the right and left gastrocnemius (GAS), soleus (SOL), TA, rectus femoris (RF) and biceps femoris (BF) muscles. We also recorded sternocleidomastoid (SCM) muscles as an index of startle reflex. For each muscle, parallel-bar EMG sensors with inter-electrode distances of 10 mm (DE2.1 sensors with Bagnoli-8 system, Delsys Inc., Boston, MA, USA) were placed on the top of the belly of each recorded muscle. Because of a large inter-subject and inter-trial variability, RF and BF were excluded from the analysis and are not further described in this article. EMG signals were band-pass filtered with cutoff frequencies of 20 and 450 Hz. The speed change for the right belt was recorded with a digital rotary encoder (2000 pulse per round) and a wheel (radius: 6 cm) attached to the surface of the belt. The resolution of the belt speed encoding (it generated 6369 pulses per second when the belt ran at 1.2 m/s) was high enough to detect the precise timing of the perturbations. All the recordings were performed in the same LabVIEW software that controlled the timing perturbations.

### Data Analysis

Data were analyzed using custom-written scripts for Matlab (The Mathworks, Inc., Natick, MA, USA). Ground reaction forces were low-pass filtered at 50 Hz using a 4th order zero-lag Butterworth digital filter. For the EMG signals, DC offset was removed and the signals were full-wave rectified. To compare the EMG signals among participants, rectified signals were low-pass filtered at 50 Hz using a 6th order, zero-lag Butterworth filter, and the amplitude was normalized to the mean maximum value during normal walking trials. We did not use maximum voluntary contraction (MVC) values as the reference of the normalization because the EMG values often exceed MVCs in the dynamic movement and reflexive burst.

To investigate proactive strategies under warned (T+) conditions, normal walking and the three catch conditions were compared. The first and second peaks of the vertical ground reaction force and the braking and propulsive peaks of the horizontal ground reaction force were analyzed. Since the most prominent difference was observed in EMG activity during the 100–300 ms immediately following right foot contact, averaged EMG amplitudes during this time window were calculated for right GAS, SOL, and TA muscles.

It is well known that reflex latency and amplitude depends on alpha motoneuron excitability. To estimate alpha motoneuron excitability for each prior knowledge condition, the averaged EMG amplitude during the 100–300 ms period that immediately followed right foot contact was calculated from the signals recorded in the corresponding reference conditions. The time window corresponds to 27–235 ms after the perturbation, given the time interval from the right foot contact and the perturbations. Startling could occur in response to the perturbations and it could potentially influence the reflex latencies (Valls-Solé et al., 2008; Ravichandran et al., 2013). To investigate the possibility of a startle effect on muscle reflex latency, we counted the number of trials in which activation of the right or left SCM was observed within 120 ms of the perturbations.

Reflexive EMG activity was observed in GAS and SOL in response to the acceleration perturbations and in TA in response to deceleration perturbations. For these three muscles, we calculated subtracted signals as a difference between the perturbed trial and the corresponding reference trial. Onset of the EMG activity was defined as the point when the subtracted signal exceeded two standard deviations of the reference signal. The analysis of EMG onset was performed with a Matlab script at first, and then visually checked and manually corrected as needed. EMG latency was calculated as the interval between the timing of the perturbation and the EMG onset. To illustrate the EMG activity patterns between different prior knowledge conditions, subtracted signals were smoothed by calculating the average amplitudes for 10-ms bins starting with the time of the perturbation.

### Statistics

To evaluate the proactive adaptation of walking strategy, peak ground reaction forces and averaged EMG amplitude measured during the 100–300 ms period that followed right foot contact were compared between normal, Catch Acc, Catch Dec, and Catch Acc/Dec conditions using one-way repeated measures ANOVA. The modest number of subject may affect statistical reliability. For easy comparison of values, partial eta squared with 95% confidence interval calculated by bootstrapping is reported as measures of effect size. *Post hoc* tests were performed if a significant main effect was observed. A two-way repeated measures ANOVA was performed to test EMG latencies among directional and temporal prior knowledge conditions. Once a significant main effect or interaction was observed, paired *t*-tests were performed as *post hoc* tests to test the effects of directional and temporal prior knowledge conditions. A significance level was set *p* < 0.05 for the ANOVAs and *p* < 0.008 for the *post hoc* comparisons after Bonferroni correction. To test the effect of temporal prior knowledge on reflexive muscle activity, the amplitude of the subtracted EMG signals were compared between D+T+ and D+T−, and between D−T+ and D−T−. These comparisons were performed over four time windows: 40~60 ms, 60~80 ms, 80~100 ms and 100~150 ms after the perturbation. The time window roughly corresponds to the latencies of short-, medium-, early component of long-, and late component of long-latency reflex. Unless otherwise indicated, data are presented as mean ± standard deviation. The number of trials in which SCM activation was observed was compared between conditions by using Wilcoxon signed-rank test.

## Results

**Figure 3:**
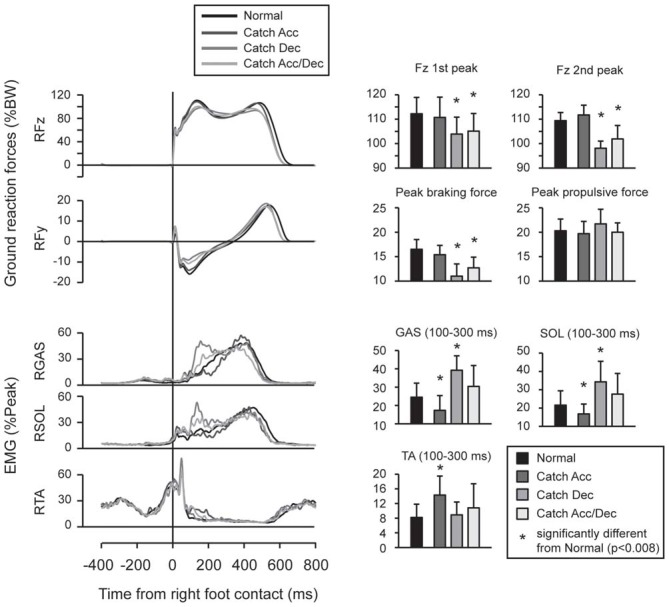
**Force and EMG modulations were observed in normal and catch trials.** When participants anticipated acceleration perturbation, Gastrocnemius (GAS) and Soleus (SOL) activity was reduced and Tibialis anterior (TA) activity was enhanced. When participants anticipated deceleration perturbation, reduced peak vertical and braking ground forces were observed which accompanied by enlarged EMG activities in GAS and SOL. In trials under which participants did not have spatial prior knowledge (i.e., they anticipate both of acceleration and deceleration perturbations), proactive modulations were similar to trials under which deceleration perturbation was expected. Error bars show standard deviations.

Feedforward adaptation was observed when the participants knew the timing of the upcoming perturbations (Figure 3). When the participants anticipated the belt would decelerate, the peak values of vertical and braking ground reaction forces were smaller than those recorded during normal walking, and were accompanied with enhanced activity in GAS and SOL. When the participants anticipated the belt would accelerate, the peak ground reaction forces were not significantly different from those recorded during normal walking, but EMG activity in TA was increased from that for normal walking.

**Figure 4:**
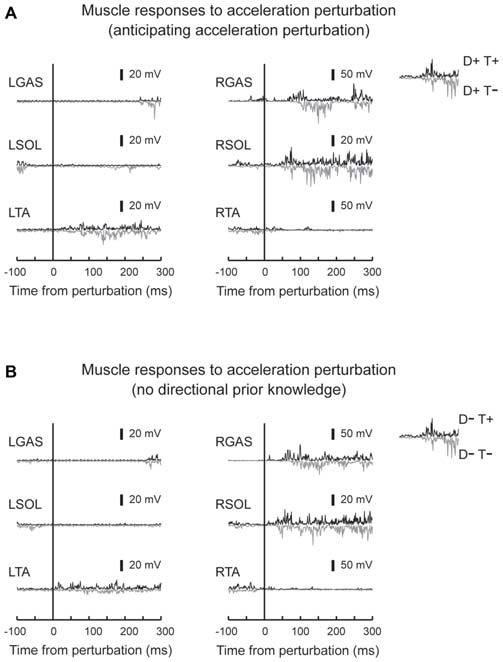
**Typical muscle responses to an acceleration perturbation.** Muscle responses when the participants had directional prior knowledge **(A)** and when the participants had no directional prior knowledge **(B)** are shown. Reflexive muscle activity was observed in the perturbed GAS and SOL muscles. Short-latency components were observed only in SOL. GAS responses shifted to earlier latencies in trials which participants knew the timing of the perturbation (downward signal). No big difference was observed in other muscles between T+ and T− conditions.

**Figure 5:**
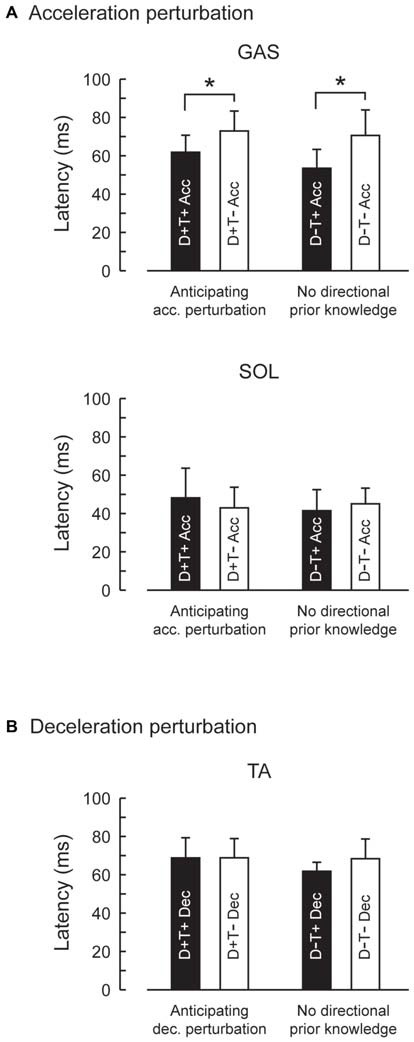
**Reflexive muscle response latencies in four prior knowledge conditions.** Latency of GAS activity evoked by the acceleration perturbation was shortened with temporal prior knowledge for the perturbation **(A)**. Prior spatial knowledge had no effect on the muscle response latency. Latency for TA activity evoked by the deceleration perturbation was not influenced by prior spatial or temporal knowledge **(B)**. Error bars show standard deviations.

EMG activity was observed in GAS and SOL in response to acceleration perturbations (Figure 4). A main effect of prior temporal knowledge on the latency of GAS activity was observed (*F*_1,10_ = 34.5, *p* < 0.001, partial eta-squared = 0.78 [95% CI : 0.67–0.90]). The *post hoc* comparison revealed that GAS latency was significantly shorter when the participants had prior temporal knowledge of the perturbation (Figure 5; D+T+: 62 ± 9 ms, D+T−: 73 ± 10 ms, D−T+: 54 ± 10 ms, D+T+: 71 ± 13 ms). We observed no main effect of prior spatial knowledge or an interaction between spatial and temporal prior knowledge conditions for GAS latency. No main effect of prior directional and temporal knowledge conditions was observed for SOL latency. A significant interaction between prior directional and temporal knowledge conditions was observed (*F*_1,10_ = 7.0, *p* < 0.024, partial eta-squared = 0.41 [0.06–0.81]), however, no significant differences were revealed with *post hoc* tests among conditions.

**Figure 6:**
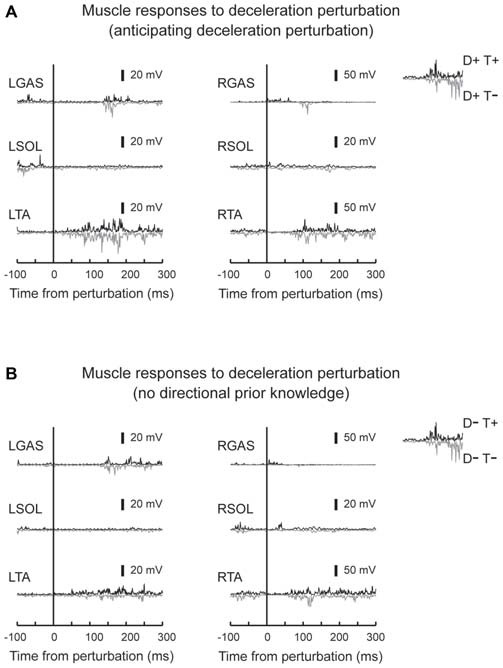
**Typical muscle responses evoked by a deceleration perturbation.** Muscle responses when the participants had directional prior knowledge **(A)** and when the participants had no directional prior knowledge **(B)** are shown. Significant muscle responses were observed in the perturbed TA muscle. Onset timing of the TA activity was not influenced by knowing the timing of the perturbation.

EMG activity was observed in TA in response to deceleration perturbations (Figure 6). Unlike GAS in acceleration perturbation trials, the timing of the TA activity in deceleration perturbation trials was not influenced by the prior knowledge conditions. No main effect of prior directional (*F*_1,10_ = 3.1, *p* = 0.11, partial eta-squared = 0.24 [0.00–0.75]) or temporal (*F*_1,10_ = 4.2, *p* = 0.07, partial eta-squared = 0.30 [0.02–0.68]) knowledge conditions or interaction between prior temporal and directional knowledge conditions (*F*_1,10_ = 2.5, *p* = 0.14, partial eta-squared = 0.20 [0.00–0.72]) were observed.

**Figure 7:**
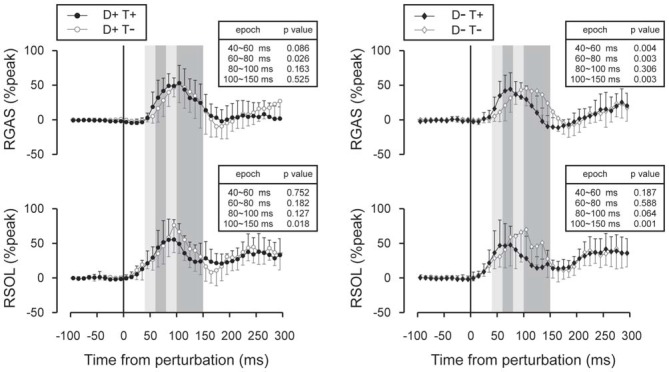
**Subtracted EMG signals in acceleration perturbation trials.** Mean and standard deviations among all participants are illustrated. GAS responses were shifted to earlier latencies with prior temporal knowledge for the perturbation. Error bars show standard deviations. The mean amplitude was compared by using paired *t*-tests for four time window: 40~60 ms, 60~80 ms, 80~100 ms and 100~150 ms.

**Figure 8:**
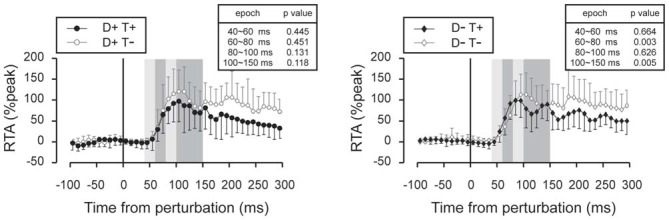
**Subtracted EMG signals in deceleration perturbation trials.** Mean and standard deviation among all the participants are illustrated. The amplitude of the long-latency component of the TA response was reduced in trials under which the participants knew the timing of the perturbation. Error bars show standard deviations. The mean amplitude was compared by using paired *t*-tests for four time window: 40~60 ms, 60~80 ms, 80~100 ms and 100~150 ms.

Subtracted binned EMG signals revealed a significant effect of prior temporal knowledge for the EMG response to acceleration (Figure 7) and deceleration perturbations (Figure 8). The GAS EMG activity pattern showed an earlier onset with prior temporal knowledge, especially between S−T+ and S−T− conditions. SOL activity amplitude was reduced during the 100–150 ms following the acceleration perturbation with prior temporal knowledge. The amplitude of TA EMG activity recorded in response to a deceleration perturbation was reduced for a long time window beginning 100 ms after the deceleration perturbation.

The SCM activity was observed in response to the perturbations (Table 1). In response to the acceleration perturbation, the number of trials in which the SCM activity was observed was different between conditions (*χ*^2^ = 9.3, *p* < 0.03). No statistically significant difference was observed in frequency of SCM observation in response to the deceleration perturbation (*χ*^2^ = 2.58, *p* < 0.46).

**Table 1:** **Number of trials in which significant sternocleidomastoid (SCM) responses were observed**.

Subject	Acceleration				Deceleration			
	S+T+	S+T−	S−T+	S−T−	S+T+	S+T−	S−T+	S−T−
1	4	5	4	3	1	2	1	1
2	0	3	4	4	1	2	2	4
3	0	5	1	3	5	5	2	4
4	1	5	4	5	1	3	3	4
5	0	3	1	3	1	1	0	0
6	4	5	5	5	1	0	1	1
7	4	5	4	4	5	5	3	5
8	3	3	3	3	0	0	1	1
9	5	5	4	5	1	1	0	0
10	5	5	5	5	4	1	0	3
11	5	5	5	4	3	2	3	3
Mean	3.9	4.5	3.6	4.0	2.3	2.4	2.0	2.9
SD	2.1	0.9	1.4	0.9	1.8	1.7	1.2	1.8

*The SCM response is an index of startling induced by the perturbations. Participants tended to show the startle response in trials under which they had no prior temporal knowledge for the perturbation (T− conditions) compared with that under which they knew the timing of the perturbation (T+ conditions)*.

## Discussion

### Proactive Strategy Based on Prior Knowledge About Upcoming Perturbations

The comparison between normal and catch trials revealed proactive adaptations when the participants knew the timing of the upcoming perturbation. Significantly reduced vertical and braking forces were observed in Catch Dec and Catch Acc/Dec conditions compared with normal walking. This may be interpreted as a cautious landing strategy, which was also reported in a previous study that investigated foot landing on a slippery surface (Marigold and Patla, 2002; Heiden et al., 2006; Siegmund et al., 2006). On the contrary, no significant difference was found in ground reaction forces between Catch Acc and normal walking. This might be because the acceleration perturbation did not interrupt the leg motion on the treadmill and thus might not affect postural balance. In fact, the participants reported after the experiment that they could automatically handle the acceleration perturbation with ease, once they were habituated. When the participants had no prior directional knowledge for the upcoming perturbation (Catch Acc/Dec), we observed feedforward adaptation which was similar to that observed in conditions where they expected a deceleration perturbation. The proactive strategy observed in the uncertain condition might reflect strategies for preparing for severe danger (i.e., the deceleration perturbation).

Differences in EMG activity patterns between normal and catch trials were observed 100–300 ms after right foot contact. Given the electro-mechanical delay of lower limb muscles, this pattern of muscle activity did not contribute to the change in the first peak of the ground reaction forces. The time window of 100–300 ms after the foot contact corresponded to the time window when reflexive muscle responses were observed in actually perturbed trials. One may think that preprogrammed EMG patterns were released in both catch and perturbed trials regardless of whether the participants were actually perturbed or not. However, the muscles responding in the perturbed trials were not enhanced as they were in the corresponding catch trials. For example, GAS and SOL, but not TA, responded to acceleration perturbations, whereas TA, but not GAS and SOL, showed enhanced activity in the Catch Acc condition. Rather, muscles that showed enhanced activities in catch trials were the antagonists of muscles that responded in perturbation trials. The enhanced muscle activities observed in the catch trials might contribute to stabilizing the ankle joint, when actually perturbed together with the muscles that responded to the perturbation. Since these analyses were performed on the last five trials of 10 experimental trials preceded by practice sessions, the observed muscle activities might be tuned for an appropriate proactive strategy for the potential perturbations (Nieuwenhuijzen and Duysens, 2007).

The significant differences between the normal and catch trials suggest that the participants had different background activity level depending on their prior knowledge for the upcoming perturbation. It also suggests that the observed raw EMG data in the perturbed trials include both proactive and reflexive components. Hence we subtracted the reference signal obtained in the catch trials from the EMG activities observed in the perturbed trials in order to extract reflexive components (see “Methods” Section for detail). We will discuss the reflexive component of EMG activities in various prior knowledge conditions based on the analyses performed on the subtracted signals.

### Reflexive Muscle Response to Sudden Acceleration and Deceleration of the Right Belt

Different patterns of muscle responses were evoked by acceleration and deceleration of the right belt. SOL and GAS responded to the perturbation in acceleration perturbation trials (Dietz et al., 1984, 1987). These activity patterns might be induced by a rapid stretching of plantar-flexors asssociated with rapid backward acceleration of the belt. The activity of the plantar-flexors might be task-relevant because they contributed to postural balance by moving the body pendulum backward and preventing a forward loss of balance (Nashner, 1976). For SOL, short and prominent bursts were observed with latencies around 50 ms (Figures 4, 5), induced by a rapid ankle dorsi-flexion in response to a backward shift of the right belt (acceleration of the belt). The short-latency reflex was not observed in GAS (Dietz et al., 1987). This might be because stretching GAS, a biarticular muscle that crosses both the ankle and knee joints, was compensated for by knee flexion that was caused by the acceleration perturbation. Some previous studies reported that SOL plays a primary role in providing postural stability at an early stance phase, and its monosynaptic reflex gain was increased during the early stance phase compared with that for GAS muscle (Moritani et al., 1990; Duysens et al., 1991).

In deceleration perturbation trials, TA activity was evoked with latencies between 60–70 ms. It is hard to assume neural pathway to mediate the TA reflex because deceleration of the belt (note that it was not a reversed movement of the belt) might not significantly stretch the TA muscle. One plausible explanation is that the TA burst was a functionally relevant medium-latency reflex (Allum, 1983). In treadmill walking, participants expect dynamic stability is achieved by the base of support moving backward and moving forward through the projection point of the center of mass. Because deceleration of the belt interrupted foot movement, a rapid TA response might be needed to bring the center of mass forward; otherwise, the participants lost their balance and fell backward.

### Temporal Prior Knowledge Shortens GAS Onset Latency Induced by the Acceleration Perturbation

Although the number of subject was relatively small (*n* = 11), our results demonstrated a significant shortening of GAS latency when the participants knew the timing of the upcoming perturbation compared with the condition where they had no prior temporal knowledge. Not only did the onset latency decrease (Figure 5), the entire EMG response pattern observed during the 70–150 ms following the perturbation in the D−T− condition shifted earlier by 20 ms in D−T+ condition (Figure 7). This finding was consistent with our previous research that demonstrated the onset latency of EMG activity was shorter when subjects were instructed that there might be a perturbation, compared with a completely naïve situation (Shinya and Oda, 2010).

What was the mechanism of the shortening of the latency? We gave temporal cue by using auditory tones, which might heighten the participants’ attention. It has been reported that attention shortens blink reflex latency when matched warning cue was given (Anthony and Graham, 1985). Brown et al. (2011) suggest that motor preparation is also an attentional phenomenon that is directed toward proprioceptive sensation. Although our results are in line with these ideas, attentional theory could not perfectly explain our results. First, temporal prior knowledge shortened GAS latency even when the participants could not anticipate the direction of the perturbation, where almost opposite EMG activity must be prepared for the acceleration and deceleration perturbation. Second, shortening of the latency was observed in GAS but not in SOL although they have similar function for postural control during walking. This discrepancy between GAS and SOL muscle might be explained by a difference in the neural pathways. The absolute value of the SOL latency (44 ms for T+ and T− conditions) suggests that the SOL reflex included monosynaptic responses. On the other hand, the GAS values (72 ms in T− conditions and 58 ms in T+ conditions) suggest that spinal and/or suprasipnal interneurons contributed to the GAS response. There is an evidence of an increased monosynaptic reflex excitability in SOL during early stance phase of hopping and walking (Moritani et al., 1990; Duysens et al., 1991). Medium or long-latency reflex components might be shortened by knowing the timing of upcoming perturbations, whereas the simplest reflex pathway, the monosynaptic reflex loop, might not be influenced by prior knowledge conditions.

Another possibility that could explain the shortening of the GAS reflex latency is the muscle length or pre-activation level could have differed between conditions. Since we did not have qualitative data that proves muscle length was consistent between conditions, it should be noted that different muscle lengths at the timing of the perturbation could lead to the observed difference in GAS latency. We consider pre-activation levels were unlikely the main explanation for the shortening of the latency, because the inter-conditional change in GAS background activity level could not explain the shortening of the GAS latency. If a large background activity in GAS was observed in Catch Acc conditions, the shortening of the GAS latency in T+ acceleration perturbation trials would be explained by the enhanced alpha motoneuron background activity. However, the results demonstrated the opposite: GAS and SOL activity levels were smaller and TA activity level was larger than in normal trials. The treadmill perturbation evoked muscle activity in SCM, which means that the effect of startle on the reflex latency should be discussed (Nieuwenhuijzen et al., 2000). Unsurprisingly, the SCM response was more frequently observed in D+T− condition (the condition where the participants did not know the timing of the perturbation) than in D+T+ condition in seven out of the 11 participants. This result clearly indicates that startle effect did not explain the shortening of the GAS latency in T+ conditions.

Some researchers reported evidence of supra-spinal neural circuits playing a critical role in a task-specific modulation of medium and long-latency response (onset latency of ~60 ms) to a perturbation. Hore and Vilis (1984) demonstrated the EMG response was appropriately modulated for a task goal in monkeys, and the modulation was abolished if cerebellar nuclei were cooled (Hore and Vilis, 1984). In human studies, cerebellar patients showed deficits in establishing a conditioned response to a perturbation on a tilting platform (Kolb et al., 2001). Hiebert et al. (1994) demonstrated that spinalized cats could not make fast and appropriate responses to unexpected changes in support levels as intact animals did, indicating supra-spinal contributions on the reflexive muscle responses. Based on the above discussion, it is suggested that prior knowledge for the timing of the upcoming acceleration perturbation enhanced activity levels of neurons in spinal cord and/or subcortical circuits that were involved in medium and/or long-latency reflex loops. These pathways likely evoked GAS activity in response to the acceleration perturbation.

Medium and long-latency reflexes are known to be flexible and induced relevant to a given task (Pruszynski et al., 2011; Pruszynski and Scott, 2012). For example, in studies investigating mechanisms of stretch reflexes in upper limbs, medium- and long-latency (50–100 ms) components in the elbow flexor muscle were enhanced when subjects were able to anticipate the timing of the perturbation (Yamamoto and Ohtsuki, 1989), or when subjects were instructed to resist to the perturbation (Lee and Tatton, 1975) while short-latency components were unchanged. Nashed et al. (2014) reported that the central nervous system is capable to produce task-relevant reflexive muscle activities in response to a perturbation with latency of 60 ms in well-trained upper limb reaching tasks. In postural perturbation tasks, functionally relevant reflexes are the first line of defense against a postural threat (Nashner, 1976). If such reflex latency can be shortened, it would allow a more secure recovery of balance after a perturbation. Our previous study suggests that the reflex latency is shortened when one has knowledge of an upcoming perturbation (Shinya and Oda, 2010). The result of the present study indicates the importance of the temporal aspect of upcoming perturbation on tuning neural excitability for modulating of reflexes.

### Limited Role of Directional Prior Knowledge on Reflex

In our investigation, we observed directional prior knowledge had no effect on muscle reflex latency. However, we cannot conclude that prior directional knowledge was unnecessary for shortening muscle reflex latency. Since we used acceleration/deceleration alternatives, the right foot was the only location of the possible perturbation. There were no options of perturbation amplitude and there were no trials with perturbations on the left foot, trunk, head or whatever. It means that even in our D− conditions, the participants had certain level of spatial prior knowledge. There are many previous studies that reported the significance of spatial prior knowledge on the modulation of long-latency reflex (Beckley et al., 1991; Timmann and Horak, 1997). The limited uncertainty about spatial element of upcoming perturbation could mask the effect of spatial prior knowledge in this study.

## Conclusion

The latency of the GAS reflex in response to a sudden acceleration of the treadmill belt was shortened from 72 ms to 58 ms if the participants knew the timing of the perturbation. In contrast, SOL onset latency (44 ms) was not influenced by directional nor temporal prior knowledge. These results suggest that the excitability in supra-spinal neural circuits that mediates medium- and long-latency reflex might be enhanced by knowing the timing of the upcoming perturbation whereas the spinal neural circuits that mediates short-latency reflex was not influenced by the prior knowledge. Although the directional prior knowledge did not influence the reflex latencies in this study, details of how central nervous system takes advantage of prior knowledge or anticipation in order to modulate reflexive postural control should be addressed in future studies.

## Author Contributions

All authors listed, have made substantial, direct and intellectual contribution to the work, and approved it for publication.

## Conflict of Interest Statement

The authors declare that the research was conducted in the absence of any commercial or financial relationships that could be construed as a potential conflict of interest.
